# Supplementary material for: A meta-analysis of the stony coral tissue loss disease microbiome finds key bacteria in unaffected and lesion tissue in diseased colonies
Source: ISME Commun. 2023 Mar 9;3:19. doi: 10.1038/s43705-023-00220-0 (PMC9998881; doi:10.1038/s43705-023-00220-0)
Supplement: Supplementary file 6 — Supplemental table legends [file 43705_2023_220_MOESM6_ESM.doc]

Any use of trade, firm, or product names is for descriptive purposes only and does not imply endorsement by the U.S. Government.

**Supplemental table legends**

**Title: A meta-analysis of the stony coral tissue loss disease microbiome finds key bacteria in unaffected and lesion tissue in diseased colonies**

**Running title: SCTLD microbiome meta-analysis**

Stephanie M. Rosales^1,2^, Lindsay K. Huebner^3^, James S. Evans^4^, Amy Apprill^5^, Andrew C. Baker^6^, Anthony J. Bellantuono^7^, Marilyn E. Brandt^8^, Abigail S. Clark^9,10^, Javier del Campo^11^, Caroline E. Dennison^6^, Katherine R. Eaton^1,2^, Naomi E. Huntley^12^, Christina A. Kellogg^4^, Mónica Medina^12^, Julie L. Meyer^13^, Erinn M. Muller^14^, Mauricio Rodriguez-Lanetty^7^, Jennifer L. Salerno^15^, William B. Schill^16^, Erin N. Shilling^17^, Julia Marie Stewart^12^, Joshua D. Voss^17^

Supplemental Table 1. **List of studies, with relevant details, included in this meta-analysis.** The studies are labeled by where they were conducted - either Florida (FL) or the United States Virgin Islands (USVI) - followed by the publication year if applicable (indicated by et al.); those that have not yet been published are labeled by the year the study began.

Supplemental Table 2. **A list of significantly differentially abundant ASVs between the vulnerable and endemic zones from field-sourced apparently healthy (AH) coral colonies.**

Supplemental Table 3.  **A list of significantly differentially abundant ASVs between the vulnerable and epidemic zones from field-sourced apparently healthy (AH) coral colonies.**

Supplemental Table 4. **A list of significantly differentially abundant ASVs between apparently healthy colonies (AH) and unaffected areas on diseased colonies (DU).**

Supplemental Table 5. **A list of significantly differentially abundant ASVs between apparently healthy colonies (AH) and lesions on diseased colonies (DL).**
