# Supplementary material for: A meta-analysis of the stony coral tissue loss disease microbiome finds key bacteria in unaffected and lesion tissue in diseased colonies
Source: ISME Commun. 2023 Mar 9;3:19. doi: 10.1038/s43705-023-00220-0 (PMC9998881; doi:10.1038/s43705-023-00220-0)
Supplement: Supplementary file 7 — Supplemental Figures [file 43705_2023_220_MOESM7_ESM.pdf]

Any use of trade, firm, or product names is for descriptive purposes only and does not imply endorsement by the U.S. Government.

Supplemental Figures

Title: A meta-analysis of the stony coral tissue loss disease microbiome finds key bacteria in unaffected and lesion tissue in diseased colonies

Running title: SCTLD microbiome meta-analysis

Stephanie M. Rosales[1,2], Lindsay K. Huebner[3], James S. Evans[4], Amy Apprill[5], Andrew C. Baker[6], Anthony J. Bellantuono[7], Marilyn E. Brandt[8], Abigail S. Clark[9,10], Javier del Campo[11], Caroline E. Dennison[6], Katherine R. Eaton[1,2], Naomi E. Huntley[12], Christina A. Kellogg[4], Mónica Medina[12], Julie L. Meyer[13], Erinn M. Muller[14], Mauricio Rodriguez-Lanetty[7], Jennifer L. Salerno[15], William B. Schill[16], Erin N. Shilling[17], Julia Marie Stewart[12], Joshua D. Voss[17]

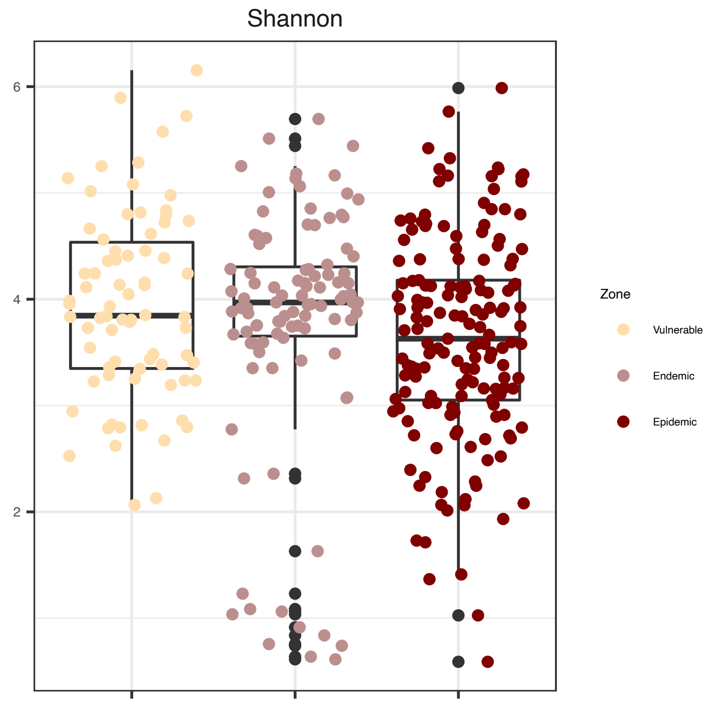

Supplemental Fig 1. **Comparison among microbial communities of field-sourced apparently healthy coral colonies across SCTLD zones (vulnerable, endemic, and epidemic)** in Shannon (alpha) diversity. In the box plot, the upper lines represent the 75th percentile, the lower lines represent the lower 25th percentile, and the mid-line represents the median. The “whiskers”

represent the data that are within 1.5 times the respective interquartile range of the maximum (upper) and minimum (lower). The black dots represent outlier samples.

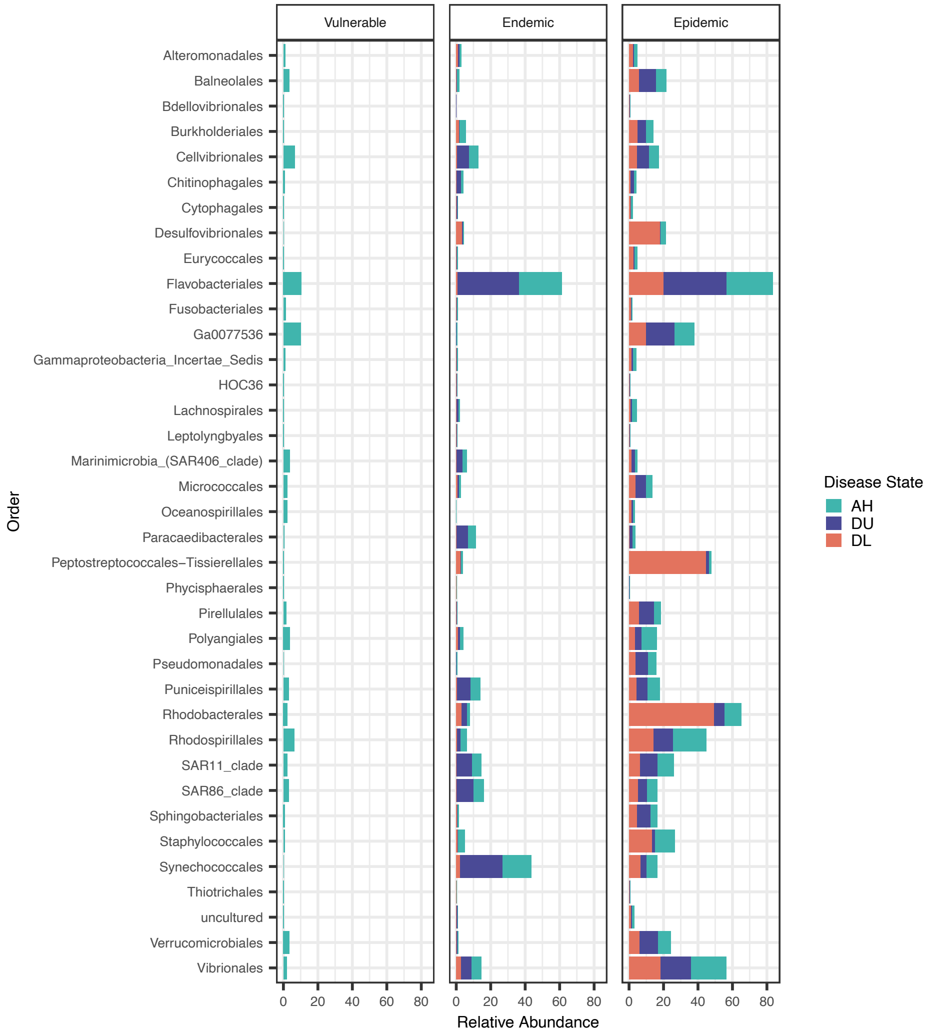

Supplemental Fig 2. **Bacteria enriched in field-sourced apparently healthy (AH) coral colonies across SCTLD zones (vulnerable, endemic, and epidemic).** The differentially abundant taxa from Figures 2 A and B among zones across health states (AH, DU, and DL) are plotted by average percent relative abundances by order. Each stacked color bar represents an SCTLD health state grouped within its respective zone. Although the statistical analysis was conducted only on AH corals all three disease states are shown.

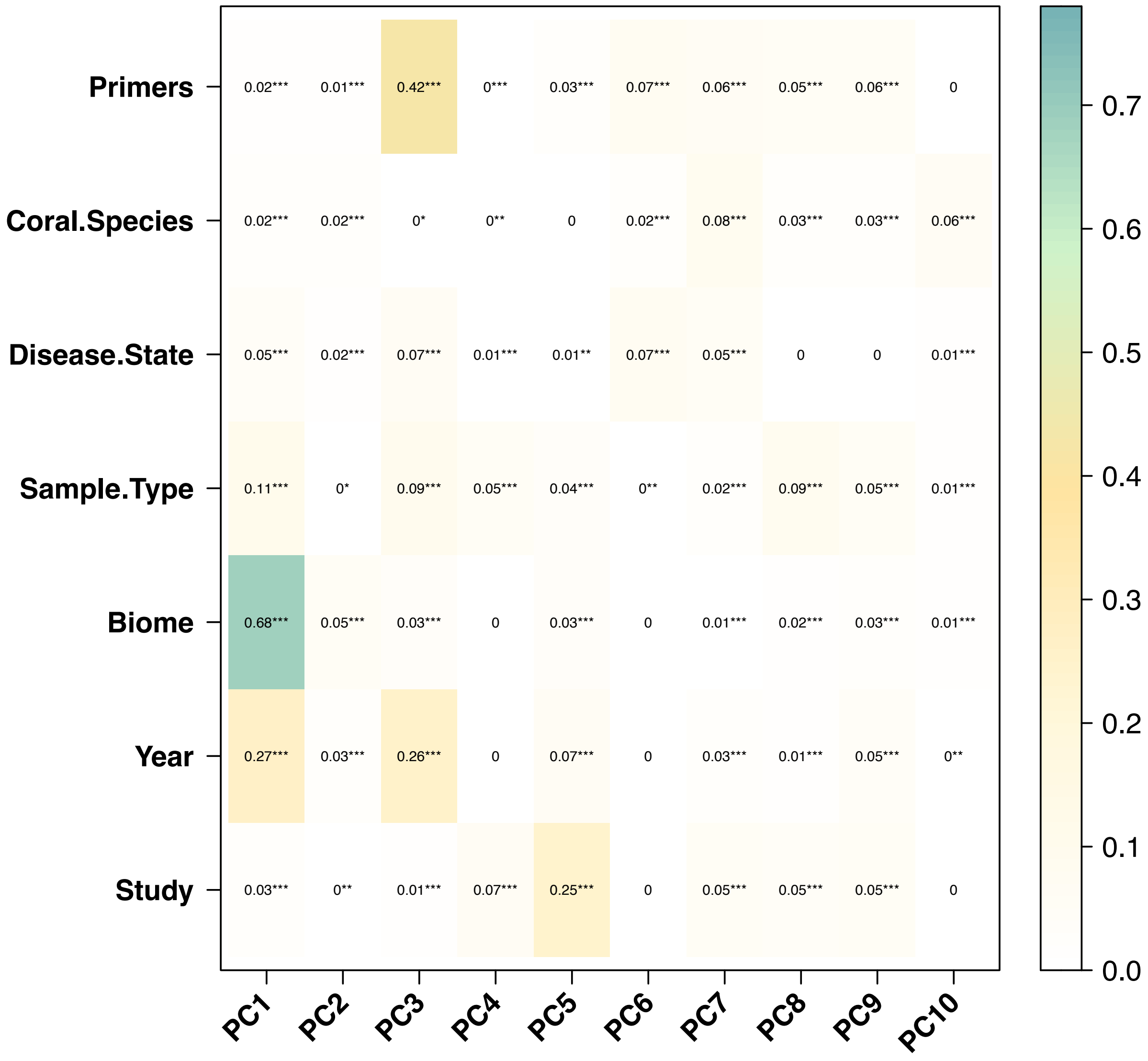

Supplemental Fig 3. **Heatmap of correlations between metadata factors (rows) and eigenvalues across principal components (PCs; columns).** The numbers and colors represent correlation values: light green represents a higher R^2 and white represents an R^2 of zero. The asterisks denote significance (****=0.0001, ***=0.001, **=0.01, *=0.05).

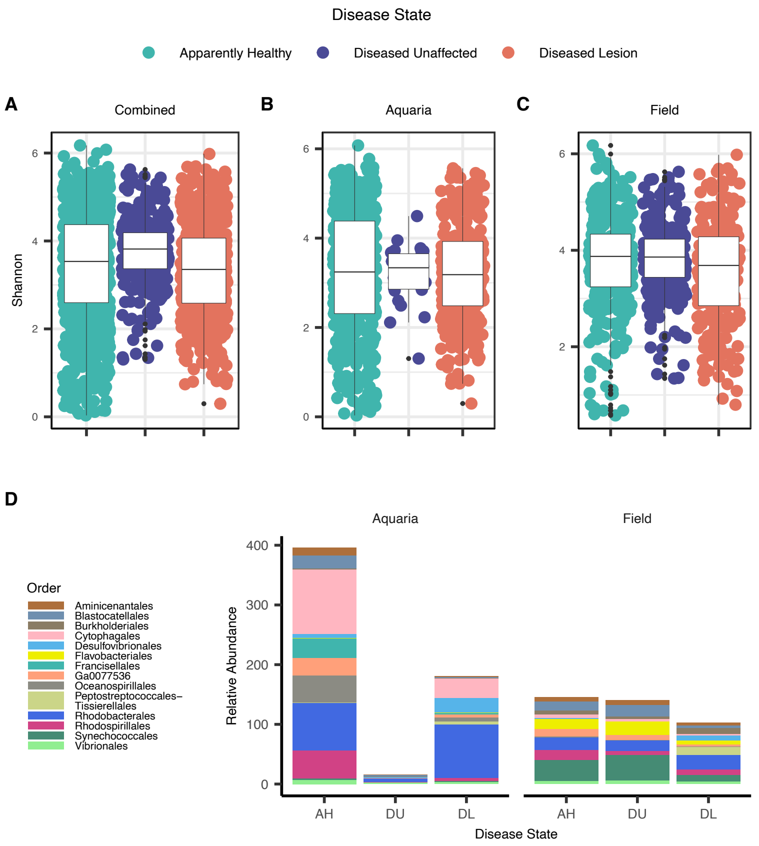

Supplemental Fig 4. **Comparisons among microbial communities of coral disease state among apparently healthy colonies (AH), and unaffected (DU) and lesion (DL) areas on diseased colonies** in Shannon diversity in (A) combined aquaria and field samples, (B) aquaria samples only, and (C) field samples only. The upper lines represent the 75th percentile, the lower lines represent the lower 25th percentile, and the mid-line represents the median. The "whiskers" represent the data that is within 1.5 times its respective interquartile range of the max (upper) and min (lower). The black dots represent outlier samples. (D) The top relative abundant amplicon sequence variants (grouped by order) by disease state for aquaria and field samples.

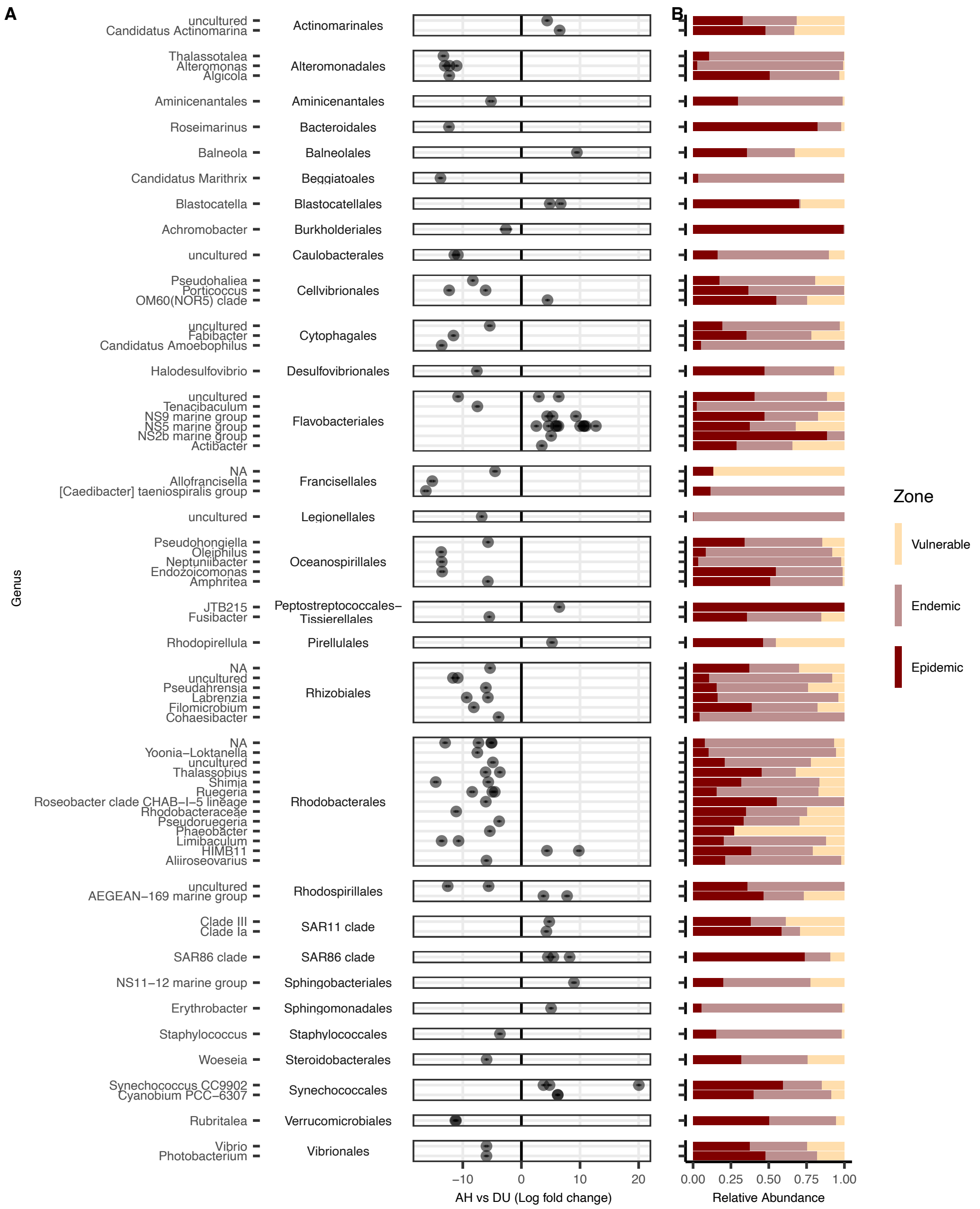

Supplemental Fig 5. **Microbial ASVs associated with unaffected areas on diseased colonies (DU) relative to zones in sediment and seawater**. Differential abundances between (A) apparently healthy (AH) vs DU. The y-axis depicts ASVs grouped by genus and then by order. Only ASVs with a padj<0.001, W statistic >90, and a log-fold change <-1.5 and >1.5 were visualized. Coral compartments (i.e., mucus, tissue slurry, and tissue slurry skeleton) were included and Acropora spp. were excluded from this analysis. (B) The relative abundance of taxa enriched in AH and DU found in seawater and sediment by zone.

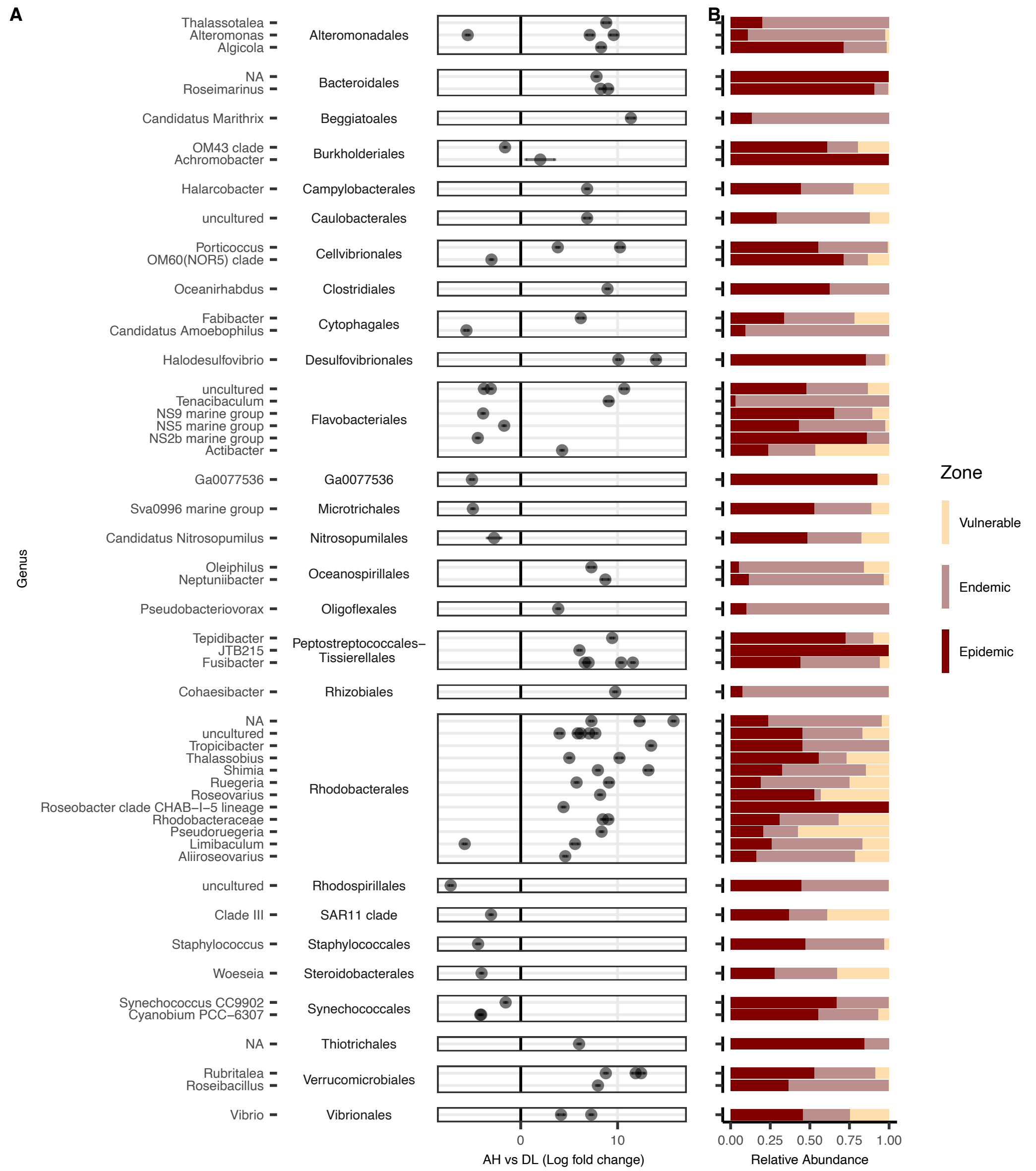

Supplemental Fig 6. **Microbial ASVs associated with lesions on diseased colonies (DL).** Differential abundances between (A) apparently healthy (AH) vs DL. The y-axis depicts ASVs grouped by genus and then by order. Only ASVs with a padj<0.001, W statistic >90, and a log-fold change <-1.5 and >1.5 were visualized. Coral compartments (i.e., mucus, tissue slurry, and tissue slurry skeleton) were included and Acropora spp. were excluded from this analysis. (B) The relative abundance of taxa enriched in AH and DL found in seawater and sediment by zone.

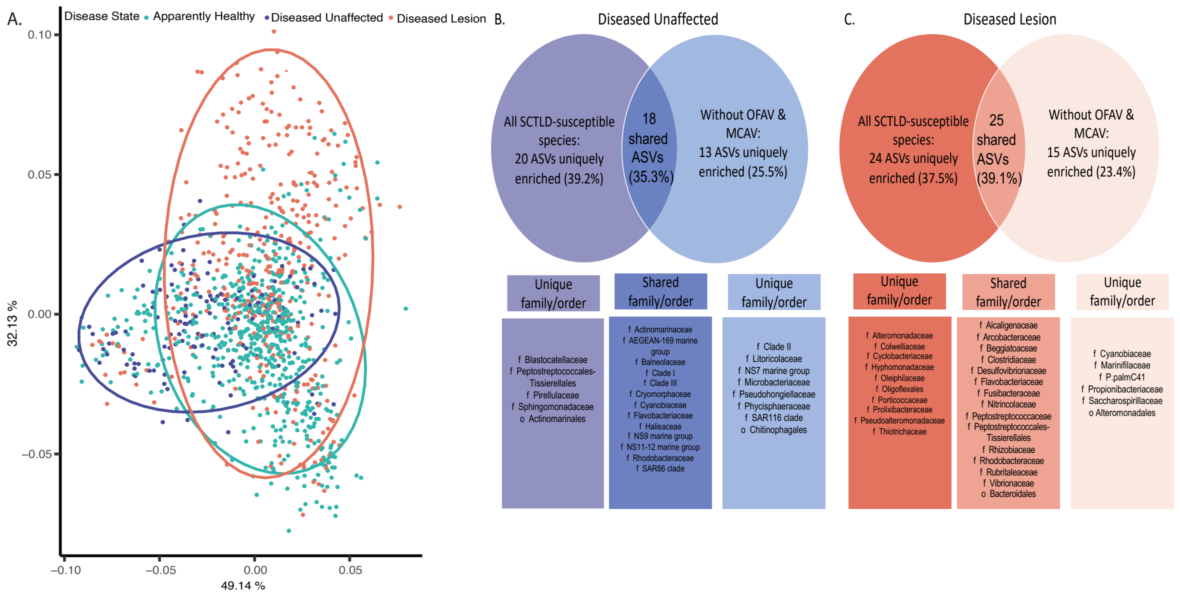

Supplemental Fig 7. **Microbial differences in coral disease state among apparently healthy colonies (AH), and unaffected (DU) and lesion (DL) areas on diseased colonies without the coral species *Orbicella faveolata* (OFAV) and *Montastraea cavernosa* (MCAV).** (A) Beta diversity of disease states without OFAV and MCAV. Venn diagrams of (B) DU and (C) DL of the number of enriched ASVs that are unique in a differential abundance analysis of all SCTLD-susceptible coral species (Figures 5 and 6), and an analysis without OFAV and MCAV, and the number of ASVs that are shared between the two analyses. The families (f) or orders (o) that correspond to the ASVs are listed below the respective group. For both differential analyses, only ASVs with a padj<0.001, a W statistic >90, and a log-fold change <-1.5 and >1.5 were included.

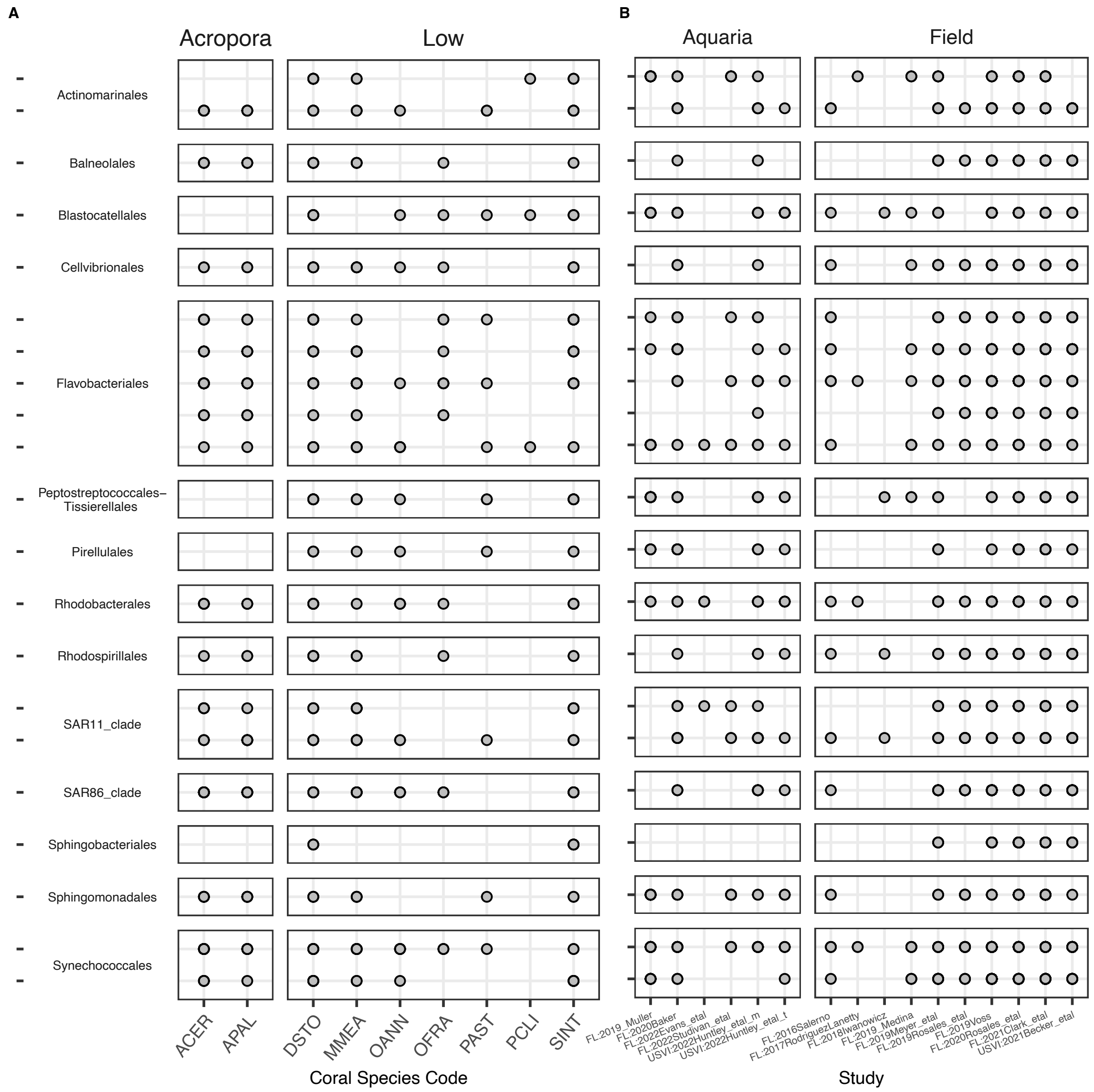

Supplemental Fig 8. **Presence/absence of amplicon sequence variants (ASVs) enriched in samples from unaffected areas on diseased colonies (DU)** in (A) coral species and (B) study. Coral species listed are those for which there were a low number of samples (n<76) relative to the other species; all differentially abundant ASVs shown were present in all sampled coral species not listed. ASVs are grouped by genus (represented by dashes) on the y-axis and then by order. Coral species codes are as follows: *Acropora cervicornis* (ACER), *Acropora palmata* (APAL), *Dichocoenia stokesii* (DSTO), *Meandrina meandrites* (MMEA), *Orbicella annularis* (OANN), *Orbicella franksi* (OFRA), *Porites astreoides* (PAST), *Pseudodiploria clivosa* (PCLI), and *Stephanocoenia intersepta* (SINT).

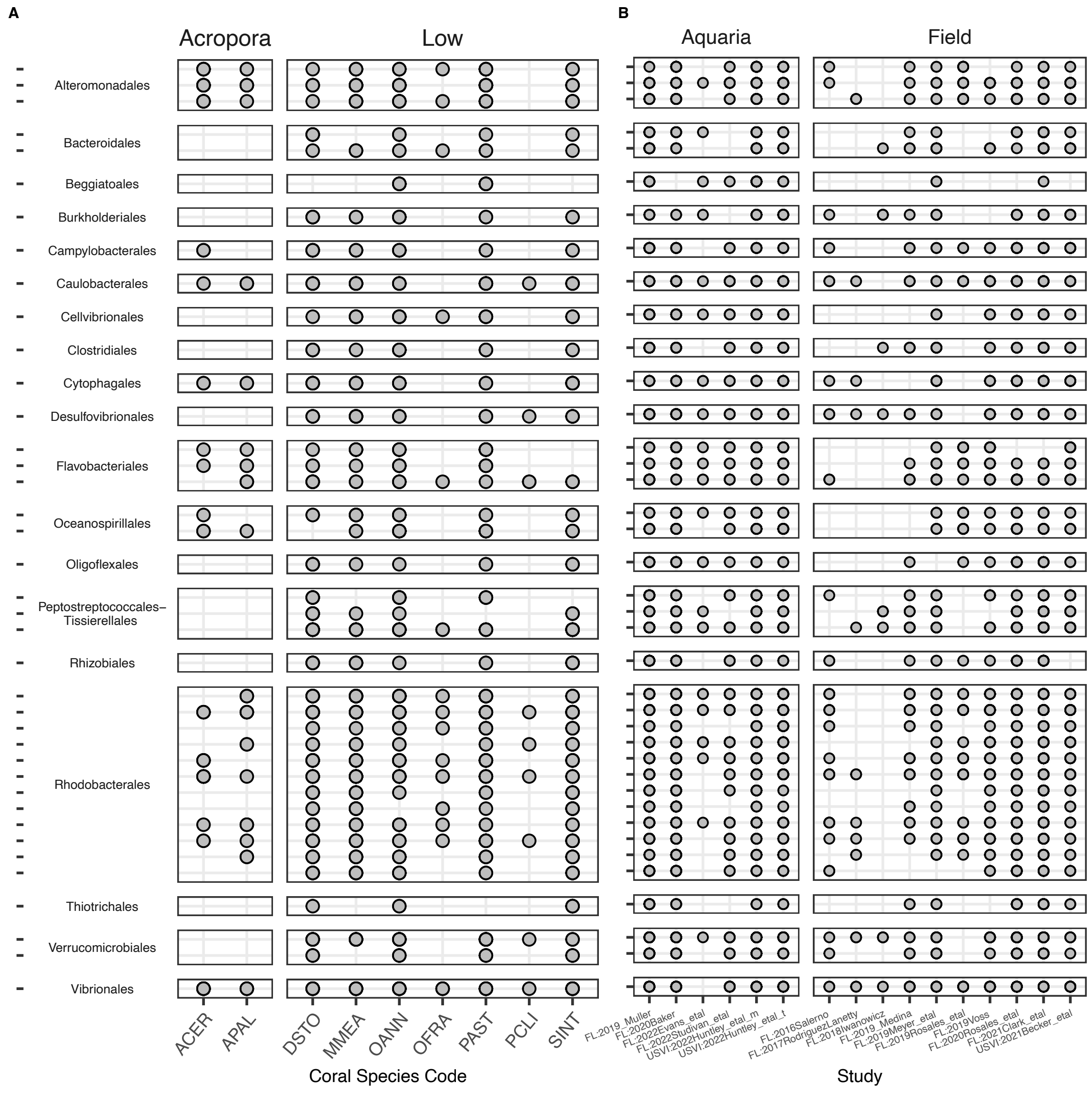

Supplemental Fig 9. **Presence/absence of amplicon sequence variants (ASVs) enriched in samples from lesions on diseased colonies (DL)** in (A) coral species, and (B) study. Coral species listed are those for which there were a low number of samples (n<76) relative to the other species; all differentially abundant ASVs shown were present in all sampled coral species not listed. ASVs are grouped by genus (represented by dashes) on the y-axis and then by order. Coral species codes are as follows: *Acropora cervicornis* (ACER), *Acropora palmata* (APAL), *Dichocoenia stokesii* (DSTO), *Meandrina meandrites* (MMEA), *Orbicella annularis* (OANN), *Orbicella franksi* (OFRA), *Porites astreoides* (PAST), *Pseudodiploria clivosa* (PCLI), and *Stephanocoenia intersepta* (SINT).

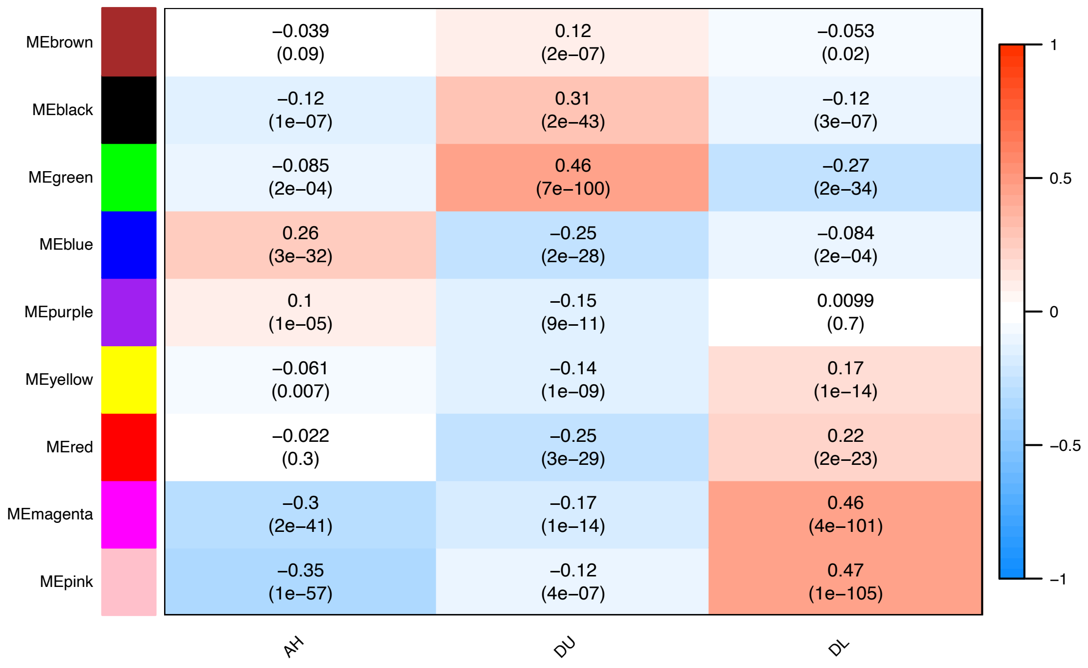

Supplemental Fig 10. **Heatmap of co-occurrence modules across disease states: apparently healthy colonies (AH), and unaffected (DU) and lesion (DL) areas on diseased colonies**. Each row is a module and is represented and labeled by a different color. The columns are the disease state. Red denotes a higher correlation of a module to a disease state and blue indicates a negative correlation from a weighted correlation network analysis (WGCNA).

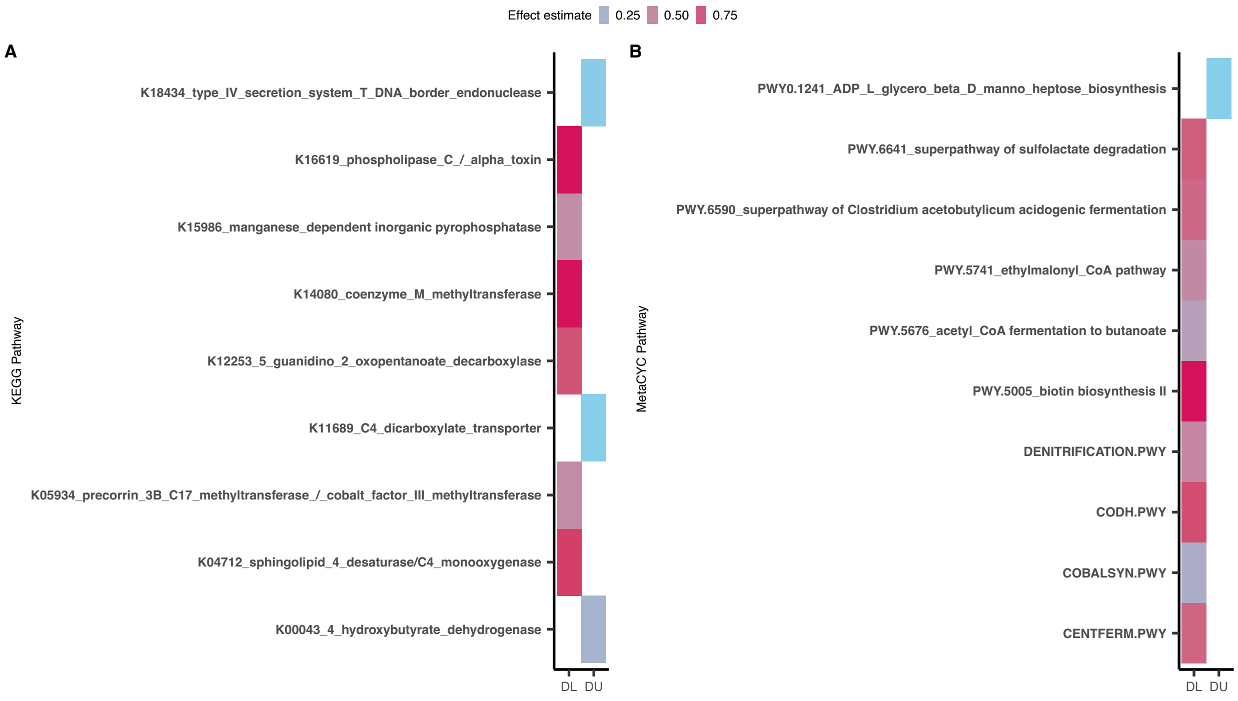

Supplemental Fig 11. **Heatmaps of the predicted function of the microbiome community in unaffected (DU) and lesion (DL) areas on diseased colonies compared to apparently healthy corals** using (A) KEGG and (B) MetaCYC pathways. The columns are the disease state and the rows represent the different pathways. Red denotes a higher effect size to disease state, white indicates no effect, and blue is an effect size between red and white.
